# Thermophysical Characterization of Efficiency Droop in GaN-Based Light-Emitting Diodes

**DOI:** 10.3390/nano11061449

**Published:** 2021-05-30

**Authors:** Tzer-En Nee, Jen-Cheng Wang, Bo-Yan Zhong, Jui-Ju Hsiao, Ya-Fen Wu

**Affiliations:** 1 Department of Electronic Engineering, Chang Gung University, Tao-Yuan City 333, Taiwan; neete@mail.cgu.edu.tw (T.-E.N.); chungeason1998@gmail.com (B.-Y.Z.); d0227103@stmail.cgu.edu.tw (J.-J.H.); 2 Department of Computer Science, National Taipei University of Education, Taipei 106, Taiwan; jcwang@mail.ntue.edu.tw; 3 Department of Electronic Engineering, Ming Chi University of Technology, New Taipei City 243, Taiwan

**Keywords:** efficiency droop, Debye temperature, electron–phonon interaction

## Abstract

An efficiency droop in GaN-based light-emitting diodes (LED) was characterized by examining its general thermophysical parameters. An effective suppression of emission degradation afforded by the introduction of InGaN/GaN heterobarrier structures in the active region was attributable to an increase in the capture cross-section ratios. The Debye temperatures and the electron–phonon interaction coupling coefficients were obtained from temperature-dependent current-voltage measurements of InGaN/GaN multiple-quantum-well LEDs over a temperature range from 20 to 300 K. It was found that the Debye temperature of the LEDs was modulated by the InN molar fraction in the heterobarriers. As far as the phonons involved in the electron–phonon scattering process are concerned, the average number of phonons decreases with the Debye temperature, and the electron–phonon interaction coupling coefficients phenomenologically reflect the nonradiative transition rates. We can use the characteristic ratio of the Debye temperature to the coupling coefficient (DCR) to assess the efficiency droop phenomenon. Our investigation showed that DCR is correlated to quantum efficiency (QE). The light emission results exhibited the high and low QEs to be represented by the high and low DCRs associated with low and high injection currents, respectively. The DCR can be envisioned as a thermophysical marker of LED performance, not only for efficiency droop characterization but also for heterodevice structure optimization.

## 1. Introduction

The increasing demand for lighting for a variety of applications such as bicycle headlights, advanced electronic displays, traffic lights, and indoor/outdoor lighting is driving the development of high-brightness nitride semiconductor light-emitting devices (LEDs), but a critical issue which remains to be resolved is the suppression of efficiency droop at high current injection levels [1,2]. Investigators have devised many ways to improve device quantum efficiency. A profound improvement in the light output power of LEDs has been obtained owing to a reduction in the threading dislocation density in the InGaN/AlInGaN multiple quantum well (MQW) active region by employing a sputtered AlN nucleation layer (NL) instead of an AlGaN NL [3]. An increase in the luminous intensity of LEDs fabricated on carbon nanotube-patterned sapphire substrates has been observed as a result of the rapid relaxation of strain in the grown GaN thin film [4]. With the use of self-organized InGaN/AlGaN dot-in-a-wire core-shell nanowire arrays, a new type of axial nanowire LED heterostructure has been fabricated with a several orders of magnitude enhancement of luminescence emission [5]. Similar observations have also been made for well-designed multi-quantum barrier (MQB) LEDs, which exhibit a higher quantum efficiency, as well as a higher temperature insensitivity, than conventional MQW LEDs [6]. The efficiency droop in nanorod-based LEDs prepared by the two-step self-limited growth method can be reduced by eliminating the quantum-confined stark effect caused by the internal piezoelectric field arising from differences in the biaxial strain between the InGaN and GaN lattice [7]. Ultraviolet LEDs with transparent conductive electrodes integrated with silver nanowires and two-step grown graphene layers have also been successfully demonstrated. The alleviation of efficiency droop, reduction in forward voltage, and enhancement of electroluminescence intensity can be attributed to the reduced heating effect in two-step grown graphene devices, caused by reduced series resistance and improved current spreading [8]. Enhancement of the external quantum efficiency of a GaN-based vertical-type LED (VLED) through the coupling of localized surface plasmon (LSP) resonance with the wave-guided mode light has also been achieved [9].

However, much effort has been devoted to unravelling the physical origin of the efficiency droop in nitride-based quantum well (QW) LEDs. In recent years, the dramatic progress in the reduction of quantum efficiency for GaN-based LEDs has brought with it many new device configurations [10,11]. They were providing profound insights into what collective processes related to the activation and inhibition of both the external and internal quantum efficiency [10,11]. Previous studies have shown that nonradiative recombination processes at high current density injection, inhomogeneous distributions of electrons and holes localized in spinodally decomposed InGaN active regions, and strong polarization-enhanced carrier overflow have important effects on efficiency droop behaviors [12,13,14,15,16,17,18,19,20,21,22], revealing two aspects of integrative analyses, i.e., current-droop (J-droop) and temperature-droop (T-droop) [23,24,25].

Indeed, the question as to what thermalization mechanisms inherently govern fermion dynamical behaviors in bosonic systems is of great importance while studying the reasons for deterioration in LED luminous efficiency. This has led to the development of electron band engineering and phonon band engineering, as well as scattering engineering for heterodevices [26]. In the past few decades, great progress has been made in advancing the understanding of the main radiative and nonradiative processes controlling the number of excess electrons and holes in nanostructures under equilibrium and nonequilibrium conditions [27,28,29]. Closely parallel to the Boltzmann transport equation (BTE), the appropriate choice of conjugated forces and fluxes, i.e., conjugated affinities and currents, leads to the well-known Onsager reciprocal relations [30]. By incorporating the Fermi–Dirac and Bose–Einstein distributions, we can build a thermodynamic equation to explore the interaction between electrons and phonons in the systems in terms of the entropy of transfer. Phenomenologically, based on Lyddane–Sach–Teller relationship, the Born–Huang equation reveals the Bloch electron dispersion, which occurs as a result of the coupling between the lattice vibration and electromagnetic waves [31]. This generally provides an important instrumental framework for understanding the electron–photon–phonon exchanging energy and momentum in the inorganic and organic condensed matter needed to produce equilibration of the heterosystem. Incontrovertibly, the energy in so-called thermophotonic systems can be transferred between the electrons/holes, the optical/acoustic phonons, the emitted photons, and the external heat and light reservoirs, suggesting that the carriers and the light may not only lose but also gain energy and momentum via the phonon absorption and emission processes [32]. It has been found that the carrier-induced transient Frenkel-pair defect generation mechanism is an important factor limiting the efficiency of InGaN LEDs, where the injected high-energy carriers induce structural instability attributable to the strong electron–phonon coupling of the excited carriers [14]. However, an interesting and unusual result has been obtained in relation to the heat-assisted emission in GaN LEDs subject to thermoelectric pumping, which is a remarkable fourfold enhancement of the light output power, indicating that electrons and holes absorbed considerable thermal energy from the lattices [33]. Apparently, from the perspective of the thermodynamics of irreversible processes, as mentioned above, the increase in radiative intensity buttresses the transfer of entropy associated with the designed nanosystems [30]. The Debye temperature is a commonly observed physical quantity which can not only be used in summing up the complete lattice dynamics of a given solid but can also provide insight into the collective processes of interacting quasiparticles [34]. It should be noted that the Debye temperature, *θ_D_*, is a function of temperature and defined only for a simple bulk substance [35]. Thus, the *θ_D_* values to be determined in the present study should be the modeled Debye temperature of the MQW/MQB device structure. Moreover, the electron–phonon coupling discussed here is of a Fröhlich type [35]. Thus, in this study, we suggest an alternative thermophysical approach to characterize the efficiency droop in GaN-based light-emitting diodes. The Debye temperature has been adopted not only to correlate the average number of occupied phonons involved in the scattering, but also to determine the corresponding relaxation time and mean free path [36]. An in-depth investigation of the thermophysical phenomena was made by observing the current-voltage characteristics and quantum efficiency (QE) of InGaN/GaN MQW LEDs at various injection currents over a wide temperature range from 20 to 300 K. The Debye temperature and the coupling coefficient were extracted. The correlation between the Debye temperature, the coupling coefficient ratio (DCR), and the QE was systematically analyzed. Further consistency checks for efficiency droop in inorganic and organic LEDs, as a function of DCR, were also made for comparison with previously published studies.

## 2. Experimental Method

**Figure 1:**
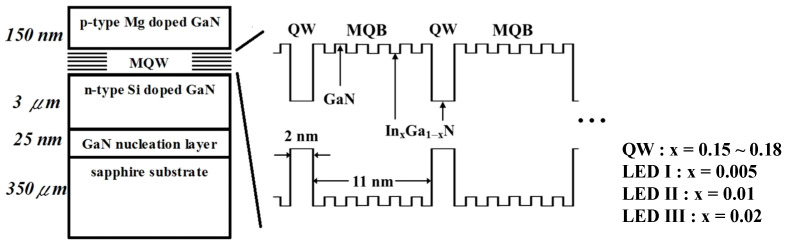
Schematic representation of the structure of the InGaN/GaN multiple quantum-well light-emitting diodes possessing different composition MQBs.

The InGaN/GaN MQW LEDs used in this work were grown on *c*-plane sapphire substrates with a 25-nm-thick GaN nucleation layer using a metal-organic vapor phase epitaxy (MOVPE) system. The wafer consisted of a 3-μm-thick n-type GaN layer, five periods of InGaN MQWs, and a 150-nm-thick p-type GaN on top. Silane (SiH_4_) and bis(cyclopentadienyl)magnesium (Cp_2_Mg) were the n- and p-type dopants, respectively, while the doping levels were nominally 5 × 10^18^ and 1 × 10^19^ cm^−3^, respectively. The MQW heterostructures of the samples were composed of five 2-nm-thick unintentionally doped In_x_Ga_1−x_N (0.15 < x < 0.18)-well layers separated by six sets of MQBs. Each set of MQBs was formed of five 1-nm-thick In*_x_*Ga_1−*x*_N layers separated by six 1-nm-thick GaN layers. We prepared three kinds of LED samples; the detailed structures of the samples are shown in Figure 1. The same material quality was maintained for all samples of the ordinary MQB structures, which were denoted LED I, II, and III. The five pairs of In_x_Ga_1−x_N MQBs had values of x equal to 0.005, 0.01, and 0.02 for LEDs I, II, and III, respectively. The current-voltage characteristics were measured using a Keithley 2430 as a current source to drive the samples mounted in a closed-cycle helium environment. The LED samples were excited using a current source operated from 20 to 60 mA under the temperature range from 20 to 300 K. The electroluminescence (EL) signal dispersed by an Acton SpectraPro 500i monochromator was detected by a Si photodiode and processed via a standard lock-in technique.

## 3. Results and Discussion

**Figure 2:**
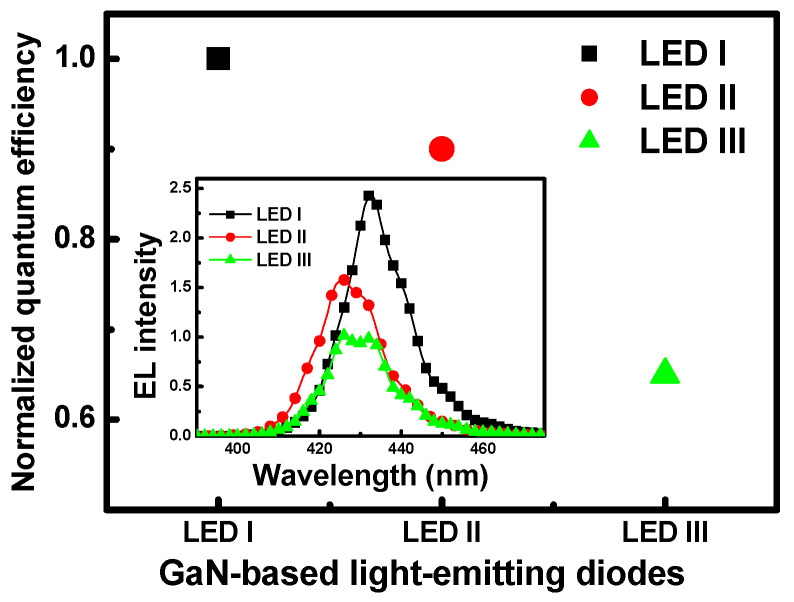
Normalized quantum efficiency for devices LED I, LED II, and LED III at room temperature and with a 20 mA injection current. The inset shows the EL spectra of samples LED I, LED II, and LED III at room temperature and with a 20 mA injection current.

The quantum efficiencies of LED I, LED II, and LED III (illustrated in Figure 2) were deduced based on the EL-integrated intensity characteristics, respectively, at room temperature from the EL spectra recorded at an injection current level of 20 mA, as shown in the inset of the figure. It is apparent from the plots that the observed EL intensity decreased with respect to an increase in the InN molar fraction (x) of the In_x_Ga_1−x_N/GaN MQBs. To exclude the influence of any other factors, all the quantum efficiencies were normalized to the observed value for LED I. The results show quantum efficiency to be consistent with our experimental observations regarding EL intensity. This phenomenon suggests that the carrier confinement depends upon the InN molar fraction for this MQB configuration, and the overflow of fewer carriers into the GaN region [37]. A comparison of changes, with an increase in the indium composition in the MQB layer from LED I to III, indicate that LED I shows a better performance of quantum recombination efficiency. The higher capture rate of electron-hole pairs in the active region, resulting from the improved quantum efficiency of LED I, would not only increase the electron concentration in the MQBs but would also reduce the carrier transport between them [38]. The excitation cross-sections of electron-hole pairs provide a very useful means of expressing the strength of a photon response to an applied injection current in the active region of LEDs [6]. Therefore, the capture and decapture cross-sections for the interaction of the carriers with the active region were statistically averaged over the cross-sections involving capture and release, respectively. The capture cross-section ratio of the electron-hole is defined as the ratio of the capture cross-section to the sum of the capture and decapture cross-sections. The capture cross-sectional ratio is an important parameter for estimating the dependency of the electron and photon relaxation process on the InN mole fraction in the samples.

**Figure 3:**
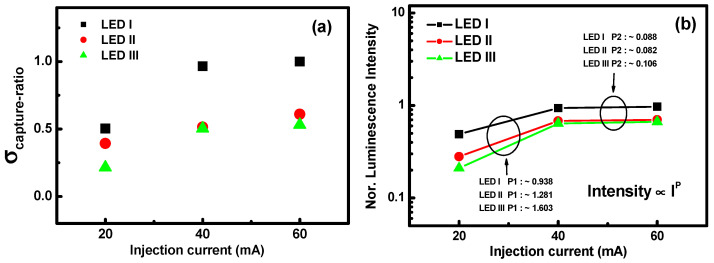
(**a**) The ratio of capture cross-section as a function of the injection current for devices LED I, LED II, and LED III; (**b**) normalized intensity characteristics of samples LED I, LED II, and LED III with different current values at room temperature.

To grasp the effect of the efficiency droop on the electron and photon relaxation characteristics, one needs to observe how the current densities start to move to higher values when the quantum efficiency reaches its peak. The increasing InN mole fraction in the MQB sample results in less carrier confinement. As far as the ability to capture carriers is concerned, it is interesting to estimate the capture and decapture cross-sections. Figure 3 presents the (a) capture cross-section ratios and (b) luminescence intensity curves obtained for the LED samples at the injection current region. The ratios of the capture cross-section dependence for a broad injection current range were extracted from the quantum efficiency. They were estimated for increasing indium concentrations from LED I to LED III and plotted in Figure 3a. It was found that all samples exhibited lower quantum efficiency with an increased InN mole fraction, as well as lower EL intensity. Furthermore, there is a dramatic increase in the capture cross-section ratios for injection currents in the region between 20 and 40 mA than current region between 40 and 60 mA in these three LED samples.From the results, we know that the larger cross-section provided by the InN composition affords a larger contribution, not only to the carrier confinement but also to the radiative recombination. Beyond this, the carrier localization and/or carrier overflow related to the high injection current region will lead to a change in the capture cross-section ratio. Figure 3b shows the normalized luminescence intensity for various injection currents ranging from 20 to 60 mA for samples LED I to III. From the results, it is evident that the normalized luminescence intensity of the LED samples is dependent on the increasing injection current level. Moreover, the observed P1 (injection current level from 20 to 40 mA) gradients of LED I to LED III were 0.938, 1.281, and 1.603, respectively, and the P2 (injection current level from 40 to 60 mA) gradients were 0.088, 0.082, and 0.106, respectively. However, with an increase in injection current level to the P2 region, there is a dramatic reduction in the normalized luminescence intensity, which indicates that there will be an increase in the Auger recombination because of the strong carrier overflow. The efficiency of droop behavior is consistent with the cross-section trend, which indicates that scattering from a continuum into bound states, included as recombination/generation terms governed by the capture times, is a significant factor in the impact of droop mechanisms.

It is well known that Auger recombination is the major cause of the droop phenomenon. To better understand how the Auger process influences the droop mechanism which inherently governs quantum efficiency, we use the McCumber–Sturge theory to assess the thermal-related electrical measurement associated with the phonon-less process and other relaxation mechanisms. According to the zero-phonon theory and the Gruneisen–Bloch relation, Debye temperature and electron–phonon coupling can be moderately accurate for most material by electrical measurement over a wide temperature range. The Debye temperature is a single parameter that contains information about vibration frequencies related to the lattice vibration of material systems. The derived function states that the resistance can be expressed as [39]

**Figure 4:**
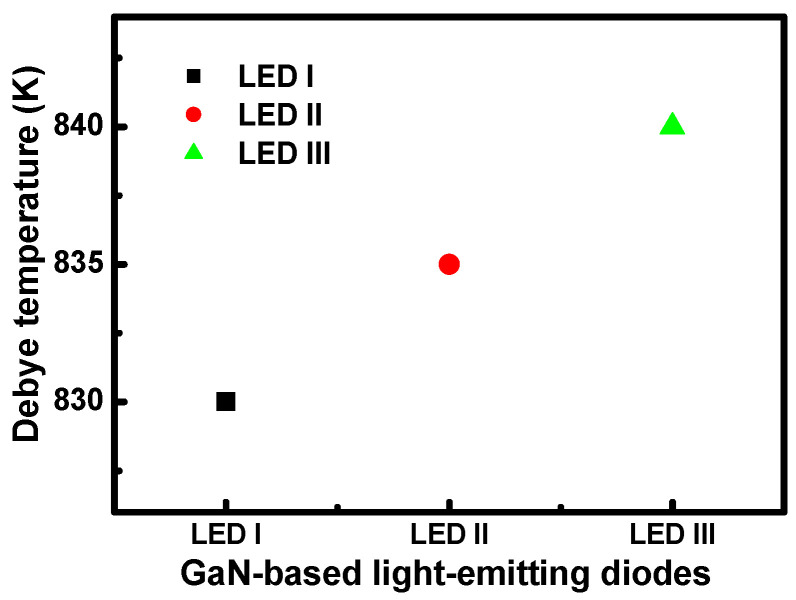
The Debye temperatures as a function of the LED structures for samples LED I, LED II, and LED III.

(1) R(T)=α(TθD)5∫0θDTx5(ex−1)(1−ex) dx,

where *R*(T) indicates the temperature-dependent resistance variation, *θ_D_* is the characteristic Debye temperature, and α is the electron–phonon coupling constant. We measured the current-voltage characteristics of the LED samples in the temperature range from 20 to 300 K. According to the Gruneisen formulation, the Debye temperature is extracted from the current-voltage analysis as a function of the LED structures, as shown in Figure 4. Consequently, examination of the terms of the temperature dependence of the resistance characteristics of LED I to LED III clearly shows that there is an increase in Debye temperature. In this study, the Debye temperature considered here is the modeled Debye temperature of the MQW/MQB structure, which is regarded as a bulk material. As the InN material component in such a structure is negligibly small, even in the device of LED III, and thus *θ_D_*, it can be determined mainly by GaN. A comparison shows that the Debye temperature of LED III was higher than that of the other LED samples, which is indicative of corresponding energy changes associated with the lattice vibration. From the results, we can see that a decrease in the capture cross-section of the electron-hole may not only cause a decrease in quantum efficiency but also an increase the in the Debye temperature of our LED samples.

**Figure 5:**
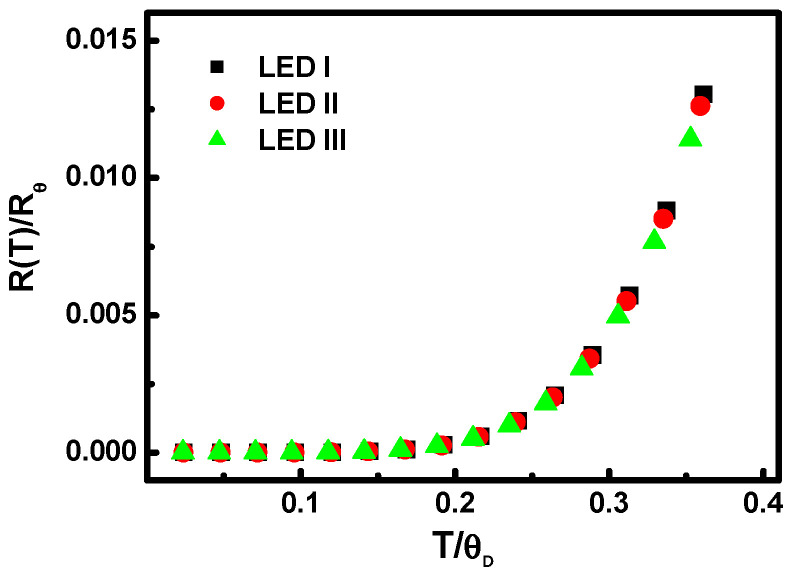
The corresponding universal curves of the Debye temperatures obtained by the so-called Gruneisen–Bloch relation from the ratio of the thermal resistance at the measurement temperature for different ratios of the cross-section for devices LED I, LED II, and LED III.

Furthermore, the Debye temperatures obtained here are corroborated by the so-called Gruneisen–Bloch relation. Take the ratio of the temperature dependence of the resistance at a specific measurement temperature over that at the corresponding Debye temperature. Figure 5 indicates that the temperature-dependent resistance can be normalized with respect to those that occur at the Debye temperature. For the ratio of cross-section dependence for MQB samples LED I to III, the Debye temperatures were 830 K, 835 K, and 840 K, as obtained by analyzing the resistance from current-voltage measurements along with temperature. The injection current level ranged from 20 mA to 60 mA. Indeed, it is significant that the observations of the resistance for LED I to III fall on the corresponding universal curves. The investigation next focused on phonon energy. The Debye temperature characteristic represents the inherent excitation dependence of electron–phonon coupling processes taking place in the LED samples.

**Figure 6:**
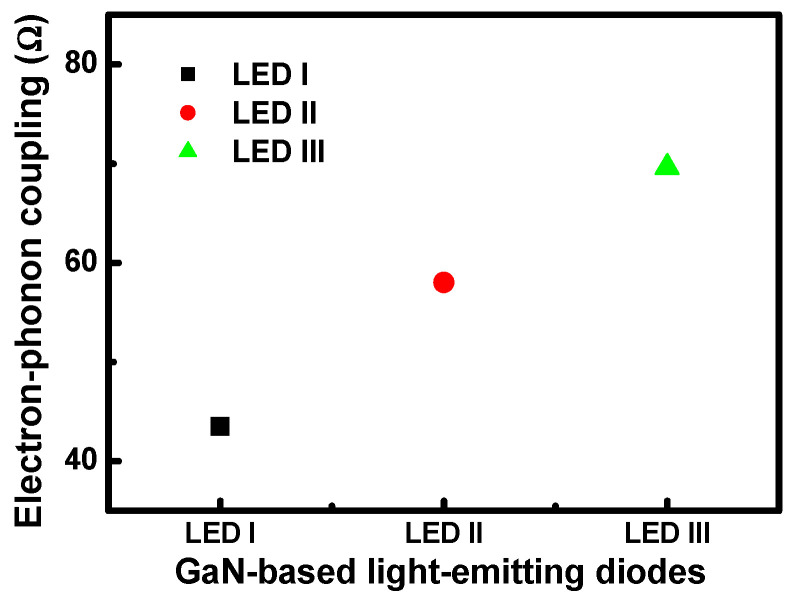
The electron–phonon coupling coefficients as a function of the LED structures for samples LED I, LED II, and LED III.

In order to more deeply understand the influence of the cross-sectional ratio on droop mechanisms, it was assumed that the electron–phonon interaction was caused by carrier-induced transient Frenkel-pair defects in the lattice thermal resistivity in these three InGaN LED samples, based on using an isotropic Debye model for the lattice waves. The increase in the electron–phonon coupling coefficients as a dependent function from LED I to III is plotted in Figure 6. It can be clearly seen that the dependence of the electron–phonon interaction coefficient in the LED samples is similar to the Debye temperature characteristics. Using the Debye model, we calculated the correlation between the thermally induced resistivity and the capture cross-sections for LED I to III, according to the current-voltage measurement, for a temperature range from 20 to 300 K. The electron–phonon interaction coefficients of these three LED samples were observed to exhibit the phonon scattering dependence as the capture cross-section ratios were increased from LED I to III. We infer from the results that carrier-induced transient Frenkel-pair defects were caused by carrier confinement and changes in the Auger recombination. Figure 6 reveals that the electron–phonon coupling shows a strong dependence on the capture cross-section ratios, while the different phonon scattering rates exhibit a relationship with the capture cross-section.

**Figure 7:**
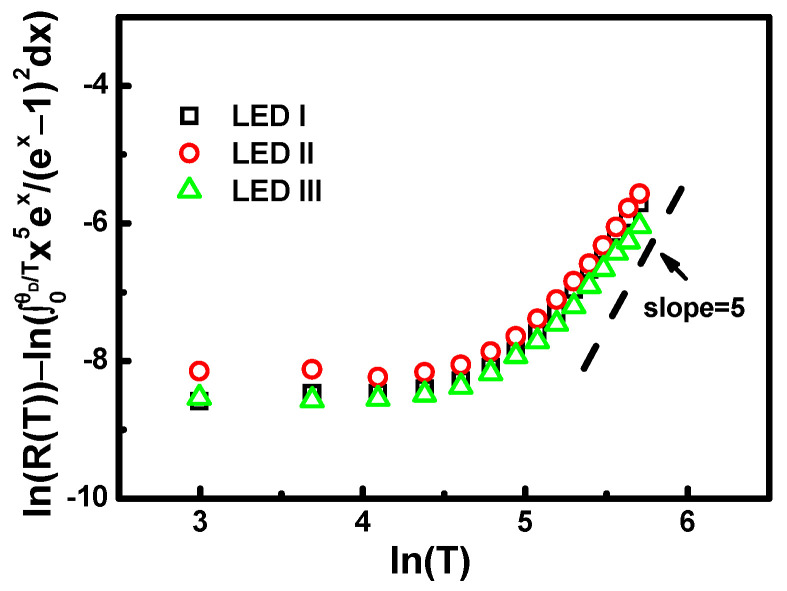
The Debye model of nonlinear analysis examining the power of the temperature through a log–log plot for associated samples.

In order to understand how the cross-section influences the photoelectric properties, the McCumber–Sturge theory is used to assess the thermal-related current-voltage observations associated with the structural configurations, and to understand the photon–phonon interactions based on particle properties. According to the theoretical calculation of the Debye model, the power–law indices for the thermal-related features were essentially five; they are indicated by the dashed lines in Figure 7. Taking the natural logarithms of both sides of Equation (1) and rearranging the terms, we arrive at the following formula:

(2) ln[R(T)]−ln[∫0θDTx5(ex−1)(1−ex) dx]=α ln(TθD)+ln α.

For a better understanding of the Debye model of nonlinear analysis, it is convenient to examine the power of the temperature through a log–log plot. According to the self-consistent formula in Equation (2), it was found that the gradients for the experimental data for the dependence of the cross-section ratio for samples after 100 K were close to 5, as shown in Figure 7. The value of the gradients, α, was dependent on the electron transition behavior. We can approximately estimate the electron–phonon coupling by using the Debye Model, but the Debye model is not consistent at low temperatures, as seen in Figure 7. The Debye model can be taken as an indication that the resistance should be proportional to the *T*^5^ law, whereas when describing acoustical phonons, analysis is restricted to low temperatures. Therefore, the resistance of some of the transition electron devices at low temperature seems to follow a *T*^2^ law [40].

**Figure 8:**
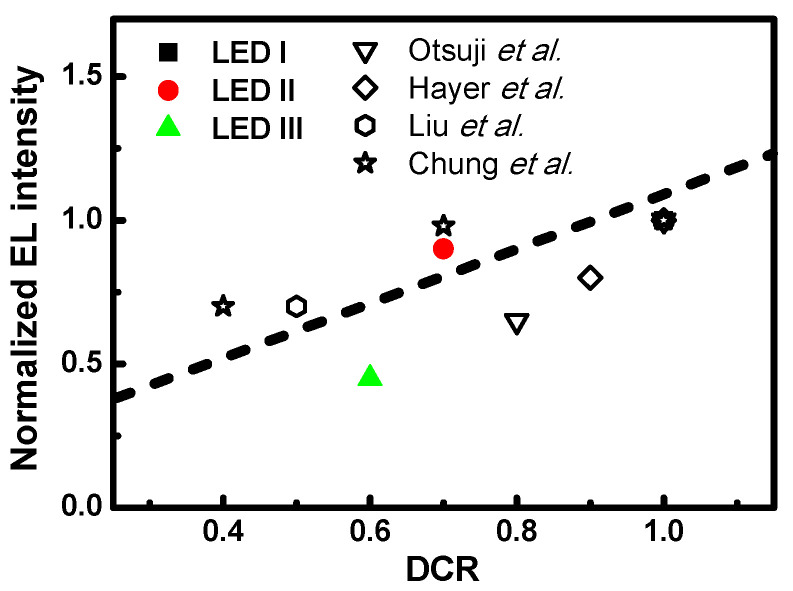
Normalized EL intensity as a function of DCR for devices LED I, LED II, and LED III.

Figure 8 shows the normalized EL intensity versus the correlation between the Debye temperature to coupling coefficient ratio (DCR). The Debye temperatures and the electron–phonon coupling coefficient ratio extracted from Figure 4 and Figure 6 by the Debye model for the LED samples show the dependence of the capture cross-section ratio under DC injection currents of 20 mA. Normalized EL intensity experiments were carried out at a temperature of 300 K. We can observe that the DCR is proportional to the EL intensity upon increasing the capture cross-section ratio for these three LED samples. A higher capture cross-section ratio leads to more energetic carriers being circumscribed in the transition zone. Based on the thermophysical theory, we know that the Debye temperature is not only correlated with the average number of occupied phonons involved in the scattering but can also perceive the corresponding relaxation time and free path. Therefore, we can observe in-depth that the carrier-induced transient defect mechanism influences device performance by observing the correlation between the Debye temperature and electron–phonon coupling. Furthermore, the above analysis provides another description of the structural configuration of the Debye temperature. The dependence of the normalized EL intensity versus DCR is also shown in Figure 8. Interestingly, the observed straight-line dependencies agree with the aforementioned expression. These findings are in agreement with the reports by Otsuji et al. regarding InGaN/GaN quantum-well diodes with and without an n-InGaN electron reservoir layer [41], as well as with Hayer et al., who examined three kinds of chemical structure of polymer light-emitting diode [42]. Other significant findings include an examination by Liu et al. of low and high power InGaN/GaN LED diodes [43] and the work of Chung et al., who analyzed organic light-emitting diodes depending on cathodes [44]. The estimated Debye temperature and electron–phonon coupling obtained from experiment data for the thermally introduced resistance from current-voltage measurement with different kinds of heterostructure configurations are shown in Table 1, respectively. Referring to the previously reported results summarized in Figure 8, we can observe that the DCR is also proportional to the normalized intensity for each series of samples. The DCR can provide a convenient way to access the energy transfer phenomena in the heterostructure devices.

**Figure 9:**
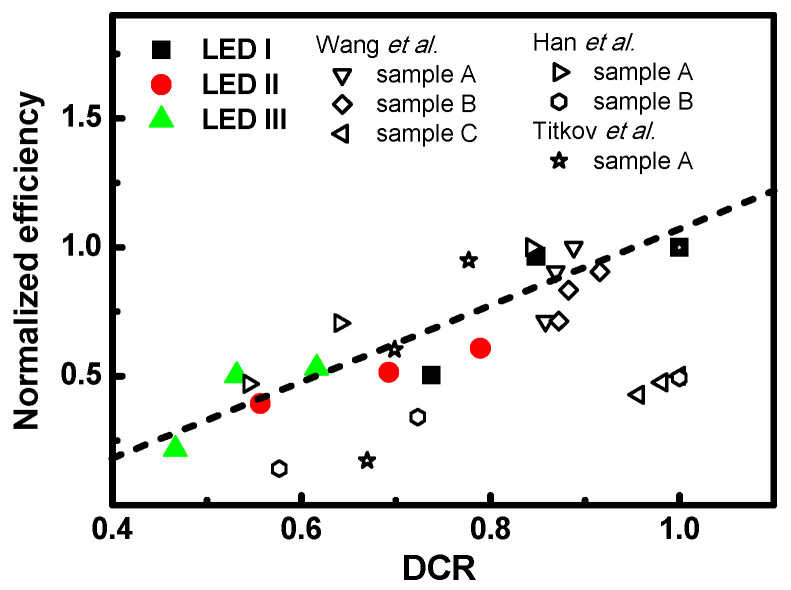
Normalized efficiency as a function of DCR for devices LED I, LED II, and LED III with different injection currents.

In order to more clearly understand the characterization of the efficiency droop in nanostructures, we can compare the normalized EL quantum efficiency versus DCR analyzed for LED sample and the dependence with the ratio of cross-section under different DC injection current from 20 to 60 mA, as shown in Figure 9. All the efficiencies were normalized to the observed injection current values at 20 mA for LED I. The DCR values extracted from the Debye temperature and electron–phonon coupling can be characterized as a function of the injection current levels. Consequently, it can be clearly seen that the dependence of the DCR on the injection current levels has a proportional relationship. Identically, the observed straight-line dependencies agree with the normalized EL quantum efficiency versus DCR analyzed expression. From the above, it can be seen that with an increase in the ratio of the cross-section in the MQB nanostructures, there will be an increase in the DCR, which will influence the average number of occupied phonons, leading to a proportional increase in the intensity. With an increase in the injection current level, if the carrier confinement in the quantum well is high, it will result in the phenomenon of Auger recombination, which would lead to the efficiency droop. Moreover, our results for thermally introduced resistance with increasing injection current levels are approximately congruent to previously published results; refer to reports of the efficiency droop phenomenon by Wang et al. for blue InGaN-GaN light-emitting diodes with different well widths [45], Han et al. for InGaN/GaN-basd light-emitting diodes [46], Titkov et al. for blue high-brightness light-emitting diodes [47], etc. The Debye temperature and electron–phonon coupling results are shown in Table 2. From the reference results reported in Figure 9, we can find out that the DCR is proportional to the efficiency for each sample at injection different current level. The dependence of the DCR on the injection current confirms that the thermodynamic processes in the particles or quasi-particles can be described by this quantitative analysis.

## 4. Conclusions

An analysis of the thermodynamic aspects characterizing efficiency droop in GaN-based LEDs was carried out. The results reveal that the figure of merit affecting the LED performance is the ratio of the Debye temperature to the coupling coefficient ratio (DCR), but the light emission efficiency behavior cannot be solely attributed to the Debye temperature or electron–phonon coupling. In an attempt to experimentally demonstrate the correlation of the characteristic ratio of the Debye temperature to the electron–phonon interaction coupling and quantum efficiency, we examined the capture cross-section ratios from high to low, corresponding to the suppression ability of emission degradation for samples prepared by the introduction of the well-designed InGaN/GaN MQB heterostructures in the quantum-well transition zone. The Debye temperatures and the electron–phonon interaction coupling coefficients were both extracted from the temperature-dependent current-voltage characteristics of InGaN/GaN LEDs. In Bose–Einstein statistics, the dependence of the Debye temperatures on the InN molar fraction in the LED indicates that the average number of phonons involved in the electron–phonon process might be modulated by the device microstructure. Furthermore, the electron–phonon coupling coefficients decreased as a function of an increase in the capture cross-section ratios, implying that the inhibition of phonon absorption facilitated the effective capture of carriers by the quantum heterowells. Our study results are consistent with other research on efficiency droop in inorganic and organic LEDs, showing that high DCR in light emissions is associated with a low current injection in response to the high QE, while a low DCR with high injection current responds to a low QE. The ratio of the Debye temperature to the coupling coefficient can thus be viewed as a thermophysical marker for the assessment of LED characteristics. A more detailed and fundamental quantitative model providing an exact description of the thermodynamic processes of the particles or quasi-particles involved in the collective redistribution mechanism will be developed in future.

## Figures and Tables

**Table 1:** Experimental data published in previous reports of thermally introduced resistance from current-voltage measurements with different kind of heterostructure configuration for estimation of the Debye temperature and electron–phonon coupling.

Samples	*θ_D_*	α
Otsuju et al. [41]		
A	800	63.8
B	750	52.2
Hayer et al. [42]		
A	415	1.4
B	960	26.8
Liu et al. [43]		
A	750	0.57
B	730	0.29
Chung et al. [44]		
A	660	2.79
B	620	1.68
C	600	1.13

**Table 2:** Data published in previous reports of thermally introduced resistance with increasing injection current levels. The estimated trend for the Debye temperature and electron-coupling is approximately congruent with our results of LED samples.

Samples	Injection Current	*θ_D_*	α
Wang et al. [45]			
A	20 mA	810	76.2
	40 mA	780	68
	60 mA	760	65
B	20 mA	800	73
	40 mA	750	64
	60 mA	740	63
C	20 mA	790	69
	40 mA	740	63
	60 mA	730	61
Han et al. [46]			
A	20 mA	860	50
	40 mA	810	40
	60 mA	800	30
B	20 mA	820	45
	40 mA	800	35
	60 mA	790	25
Titkov et al. [47]			
A	1 mA	820	70
	10 mA	815	60
	100 mA	800	30
